# A year of living distantly: global trends in the use of stay-at-home orders over the first 12 months of the COVID-19 pandemic

**DOI:** 10.1098/rsfs.2021.0041

**Published:** 2021-10-12

**Authors:** Toby Phillips, Yuxi Zhang, Anna Petherick

**Affiliations:** Blavatnik School of Government, University of Oxford, Oxford, UK

**Keywords:** COVID-19, pandemic, policy, lockdown, government, subnational

## Abstract

During the first year of the COVID-19 pandemic, non-pharmaceutical interventions (NPIs) were the main pillar of defence to protect human society against the virus. While a variety of modelling studies try to quantify the effects of NPIs, this paper investigates when and how national and subnational governments have taken actions. We observe longitudinal changes in the global pattern of policymaking to combat the COVID-19 pandemic, with a particular focus on stay-at-home orders. Drawing on data from the Oxford COVID-19 Government Response Tracker, we show several important trends. First, while national governments exhibited a strong alignment in policy settings initially in March and April 2020, their cross-country policy heterogeneity has grown since May 2020, although countries within global regions continue to display similarities in their approaches. Second, most governments that have implemented multiple stay-at-home orders over the course of the pandemic have become less sensitive to case levels (insofar as they implement subsequent restrictions at progressively higher case levels), apart from a small number of contrast cases which have mostly eliminated domestic community transmission. Third, pandemic policies are increasingly specific to subnational levels, and there is often significant heterogeneity with regard to policy approaches even within the same country.

## 1. Introduction

Global transmission of the SARS-CoV-2 virus was first detected in January 2020, when cases outside of China were reported in France, Japan, Thailand, the USA and several other countries. Since then, over the course of the pandemic, almost every country in the world has experienced one or more waves of epidemic community transmission [1–3]. High levels of COVID-19 cases have threatened to overwhelm healthcare systems in many countries, and in some cases have collapsed them, compounding the infection fatality rate of the virus and causing high excessive mortality [4,5].

In this context, especially in the period prior to national COVID-19 vaccination programmes, governments have relied heavily on non-pharmaceutical interventions (NPIs) to counter virus spread. While the effectiveness of NPIs has been a topic of considerable research effort [6–11], the patterns of their adoption have received somewhat less attention.

Here, we review the global trends in NPI policies to March 2021, the first year of the pandemic. Moving through those first 12 months, we draw out three macro patterns in the enactment of government policies against COVID-19. Focusing first on the period January–April 2020 and the months immediately after, we describe the sudden and widespread initial implementation of NPIs, within which stay-at-home orders—which epitomize the toughest trade-offs that policymakers have had to make between public health and human rights—were usually among the last closure and containment policies to be enacted by governments, even though the overall ramp up of stringency was rapid. We then describe the period of loosening of NPIs and subsequent reimposition of stringent policies, when substantial global divergence emerged. This divergence was far from random with respect to stay-at-home orders. Indeed, the second macro pattern that we call attention to is that the point when countries reimpose stay-at-home orders correlates with their prior experience of SARS-CoV-2 community transmission. Third, expanding our description of divergence, we show that over time, it has extended within countries as well as between them, as different subnational jurisdictions within a country have, over time, tended to approach the pandemic differently. We also observe that this subnational divergence is not consistent with the logic of the most stringent approach within a country being applied where transmission rates are worst.

## 2. Non-pharmaceutical interventions

At their most severe, the use of NPIs has taken the form of stringent ‘lockdown’ policies that dramatically restrict the levels of interaction between people in the community, and thus, the opportunities for transmission of the virus. We mostly focus on the varied use of stay-at-home orders as the most extreme form of NPI—literally confining the population to their own homes, usually with limited exceptions for essential excursions outside. We use data from the Oxford COVID-19 Government Response Tracker (OxCGRT) [12], which records daily policy settings for almost every country in the world across 20 NPIs, including stay-at-home orders.

Before exploring the use of stay-at-home orders, it is worth noting that the World Health Organization (WHO) has regularly and frequently cautioned against the long-term use of these measures. From the early days of global transmission, before even the official pandemic declaration on 11 March 2020, the WHO stressed the importance of what has become known as the ‘test, trace, isolate’ strategy of proactive disease surveillance, contact tracing and effective quarantine of potentially infected people [13,14]. By April 2020, the WHO's COVID-19 strategy declared ‘For countries that have introduced widespread physical distancing measures and population-level movement restrictions, there is an urgent need to plan for a phased transition away from such restrictions' [15]. When the WHO published this guidance, almost 70% of countries had compulsory stay-at-home orders or curfews in place.

Thus, in the WHO's eyes, the toughest NPIs are a stop-gap measure in the absence of effective pharmaceutical interventions. Many countries did transition away from such restrictions—at least temporarily—but stay-at-home orders have remained a core feature of the global response. At any point in time between March 2020 and March 2021, over one-quarter of countries in the OxCGRT dataset, which covers around 190 countries, have had compulsory stay-at-home orders. And more recently from January to March 2021, this has been around half of all countries at any point, despite the approval of several effective vaccines in most countries.

The rapid development and approval of COVID-19 vaccines have given countries the hope to avoid highly stringent NPIs in the future. However, in all but a few countries, the roll-out of vaccination campaigns in 2021 has been slow. Early vaccine success stories like Israel and the UK have proven hard, at least initially, to replicate in large, developed countries, such as Canada or Australia [16]. For low- and middle-income countries, they also face additional challenges to vaccine roll-out, given their typically weaker public health system infrastructures and a significant shortfall in vaccines allocated through the COVID-19 Vaccine Global Access project (COVAX) [17]. Even in some countries with relatively good vaccine supply, misinformation and low levels of vaccine acceptance may make it difficult to achieve herd immunity [18]. Meanwhile, uncertainty remains as to the effectiveness of approved vaccines against the spread of new variants. NPIs, even including stay-at-home orders, are therefore likely to continue as a core policy options of defence against COVID-19 for the remainder of 2021 and into 2022 [19–21].

## 3. From initial convergence to gradual divergence

### 3.1. January to April 2020: the early ramp up

During the initial months of the COVID-19 pandemic, countries demonstrated a remarkably similar pattern of policy selection. This similarity is observed in terms of the timing of action, the speed of escalation, the level of strictness they eventually arrived at and the sequencing of policy decisions.

The OxCGRT systematically records government policies across 20 different dimensions, such as school closures, contact tracing, stay-at-home orders and income support. Combinations of policy indicators are also aggregated into several indices between 0 and 100, such as the stringency index, which reflects the overall strength of closure and containment measures [12]. These data show a global inflection point in policy response around the third and fourth weeks of March 2020, just after the WHO pandemic declaration. At the beginning of March 2020, 90% of countries had a stringency index value in the range 0–25. By the end of March, 90% of countries were in the range 45–95. This alignment in enactment of relatively stringent policies occurred regardless of local conditions and transmission rates within countries—most countries had not recorded a single COVID-related death at this point.

Going beyond the broad convergence in overall policy strength in March and April 2020, we also observe striking similarity in the *sequencing* of individual policies: a country is more likely than not to have started with a public information campaign and some level of international travel controls in their first weeks of responding to COVID-19. Next steps often include establishing testing regimes, and closures of schools and public events. Broad stay-at-home orders, economic support and mask-wearing mandates most often come last in a country's response, some weeks or months after the first policies were implemented [12].

Retrospective studies of this period have generally found that both the overall stringency of NPIs and the speed of implementation contribute to reducing cases and deaths [1,12,22–23]. At the very least, the initial global response managed to steady what was, at that time, a rapidly accelerating pandemic. For the five weeks from 26 February to 1 April 2020, the rate of global transmission increased from 5700 new cases per week, to 479 000 (an exponential growth rate of ×2.4 per week). For the five weeks after 1 April 2020, the world was at a steady plateau of around 500 000–600 000 new cases per week (although these early numbers come with significant uncertainty due to wide variation in testing capacity across countries). This steady plateau, however, did not last.

### 3.2. May 2020 onwards: policy roll-back and divergence of national-level responses

After the rapid and globally synchronized increase in policy stringency, many countries started to ease their policy response after April 2020. This coincided with a brief plateau in global pandemic transmission, described above. Indeed, across the three months April, May and June 2020, almost every country—151 of the 186 assessed—had periods of less than 50 reported cases per day. For some countries—such as Switzerland, Malaysia and Israel—this was because an initial outbreak was brought under control. But for 72 of these countries—such as Georgia, Uganda and Jordan—they still had not recorded a domestic outbreak above 50 cases per day by June 2020, and chose to ease their precautionary policies implemented during the global convergence in March and April.

Unlike the mid-March alignment in stringent policy settings, countries loosened their policies at different times. We observe a general divergence in the OxCGRT data as each country determined its own approach. At the beginning of April 2020, the distribution of countries' stringency index levels had an interquartile range of 17 points (on the index's 100-point scale). By mid-June, the interquartile spread between countries had widened to 30 points, which persisted for many months. This divergence in country-level NPI responses coincided with the end of the short-lived plateau in COVID-19 case numbers. Into mid-June 2020, there were 1 million new cases per week, and this had risen to 4 million per week by November 2020.

As countries started diverging in their responses and (generally) loosening their policies from April 2020 onwards, we continue to observe similarity in how policies are sequenced. Social restrictions and closures are the first to be removed, whereas public health policies (such as contact tracing or mask-wearing) and economic support measures are much stickier: they persisted for long periods without removal [12].

### 3.3. Regional patterns emerge

In addition to these similarities in the sequencing of individual policies, we observe regional patterns in countries' tendency to have the harshest closure and containment policies in place. Looking further into the use of stay-at-home orders helps illustrate the ways in which different approaches were dominant in different regions.

The OxCGRT dataset records stay-at-home orders on a four-level ordinal scale from 0 to 3 indicating the strictness of the policy, from which we use levels 2 and 3 (indicating a *required* as opposed to a *recommended* policy). Additionally, the dataset includes a second binary variable to reflect whether stay-at-home orders apply country-wide or only in a targeted geographical region. For the purposes of the analysis in this section, we look simply at whether there is a compulsory stay-at-home order or curfew (with or without exemptions) in place anywhere in the country. This allows useful analysis, but we acknowledge the limitations of such an expansive definition: when we discuss ‘stay-at-home’ orders, this broad category includes the reported militarized lockdown and arbitrary arrests in Rwanda, to a night-time curfew (18.00–6.00) in Zimbabwe [24,25].

**Figure 1.**
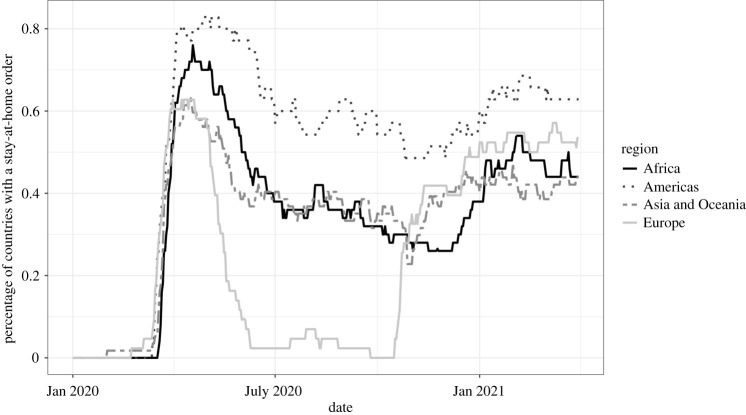
The proportion of countries implementing stay-at-home orders varied significantly between regions over the middle months of 2020. Note: countries are grouped together based on the UN Statistics Division regional groupings.

By the end of April 2020, a majority of countries in all regions had implemented stay-at-home orders, as seen in figure 1. As the year progressed, moving into the Northern Hemisphere summer, many countries began to remove their stay-at-home orders. News reporting from this period suggests that this easing was partly due to epidemiological success [26], but for many temperate Northern Hemisphere countries, also due to expectations about the seasonal nature of the virus, and a hypothesis that it would be less infectious in warmer climates [27]. If the initial March and April response was one of the countries looking globally and jumping on the bandwagon, the later use of stay-at-home exhibits more regional tendencies.

As figure 1 shows, the initial release of nationwide stay-at-home orders was most pronounced in European countries, where the proportion of countries with a stay-at-home order in place fell from over 60% (*N* = 43) in April to 5% (2 countries) in mid-June–July, and briefly hit 0 in early October. This occurred alongside a broader ‘re-open EU’ initiative the European Commission launched at the beginning of June 2020 [28,29]. For comparison, stay-at-home orders remained in place in over 30% of countries in Asia and Africa in the period July–October and in around 60% of countries across the Americas. The European summertime avoidance of stay-at-home orders did not last, however: during the last 15 days of October 2020, a dozen countries across Europe implemented stay-at-home orders as case rates within the region began to rise in the lead-up to their winter, bringing the use of restrictions across the region more in line with the rest of the world (figure 1) [30].

In some Latin American countries, we observed a different trend. Here, the stay-at-home orders took on a unique flavour: cyclical exemptions. During their most stringent lockdowns, Panama, Colombia and Peru introduced a gender-based rotation system whereby only women were allowed out on certain days of the week and only men on the other days. In Bolivia and Honduras, exemptions to leave the house were granted on different days of the week based on identity card numbers, whereas in Ecuador, Costa Rica and Paraguay, it was based on vehicle licence plate numbers [31–33]. These types of policies are not entirely new: cities such as Ulaanbaatar, Paris, Beijing and many Latin American cities have addressed air pollution by only allowing certain licence plate numbers to drive on certain days [34,35]. However, the widespread application to manage COVID-19 stay-at-home orders is a regional phenomenon largely unique to Latin America.

## 4. Countries’ threshold for imposing harsh lockdown policies changes over time

Taking forward our exploration of stay-at-home orders, we identify a global pattern in decision-makers' apparent sensitivity to case numbers over time across multiple outbreaks or waves of transmission. In this analysis, we treat confirmed, daily, new COVID-19 cases as a salient signal to policymakers about current epidemiological risk. We look at the subset of countries that implemented more than one stay-at-home orders over the period January 2020–March 2021. For most countries, we find that subsequent lockdowns after their first stay-at-home order were enacted at much higher case levels than the first stay-at-home order.

Researchers and modellers have proposed theoretical reasons why it might be advantageous to delay the imposition of stringent lockdown measures and allow a virus to spread: either to learn more about a novel virus [36] or to split an inevitable large peak of hospitalizations into two smaller peaks [37]. But we have not observed decision-makers deliberately pursuing either of these theoretical strategies. Policymakers themselves have proposed delaying or minimizing strict lockdowns because of concerns about behavioural fatigue, low compliance, economic impacts or human rights [38–40]. For example, in mid-September 2020, Prime Minister Boris Johnson warned the second lockdown in the UK would be ‘disastrous’ for the economy and had committed to doing ‘everything in our power’ to prevent it [41,42]. Health system capacity and elasticity have also been cited by policymakers when justifying the timing of subsequent stay-at-home policies [43,44].

Our analysis of the OxCGRT data is consistent with a ‘desensitization’ to case numbers, or perhaps increasing concern for the economic impacts of lockdown relative to epidemiological concerns: as the pandemic continues to progress it takes a higher level of COVID-19 cases to elicit a similar response from national policymakers (in most contexts). For example, the first stay-at-home order in the Philippines was implemented on 15 March 2020 when the country recorded 19 cases per day. The second was in June at around 560 confirmed cases per day. The third was in October at around 2500 known cases per day. Similar patterns exist in most countries, from the UK to Lebanon.

**Figure 2.**
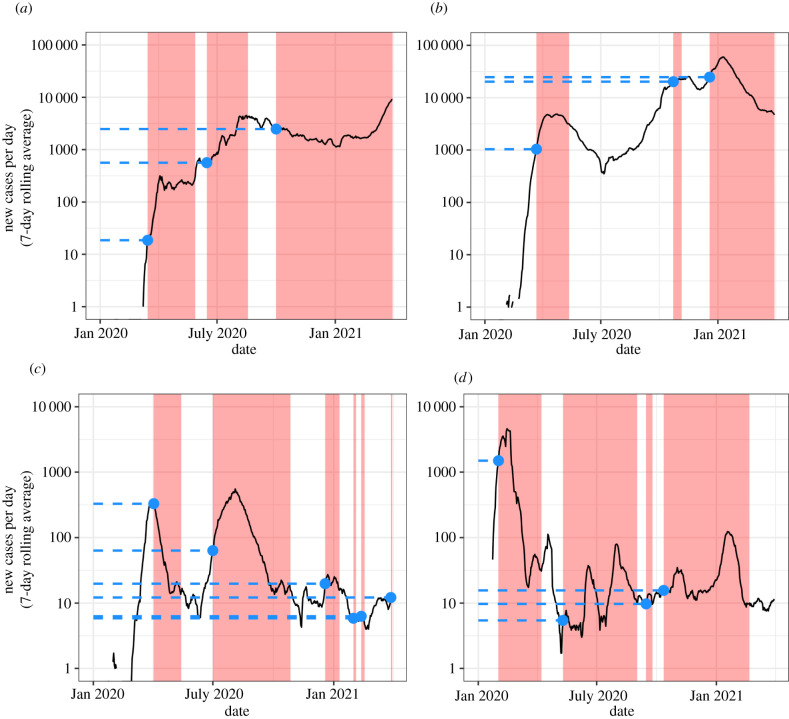
Reported case levels at the point of implementing stay-at-home orders in four countries. Note: the shaded red area represents a period during which there was a stay-at-home order or curfew in effect at some place in the country. The dots mark the number of new cases per day at the point when each successive stay-at-home order was implemented. (*a*) Philippines, (*b*) UK, (*c*) Australia and (*d*) China.

A small number of countries exhibit the reverse tendency: implementing stay-at-home policies at progressively earlier points in subsequent waves of transmission. For instance, Australia implemented its first stay-at-home order when it had around 330 confirmed cases per day. After dramatically reducing transmission, Australia subsequently reimposed stay-at-home orders when transmission reached 60, 19 and 5 recorded cases per day. Figure 2 shows these patterns for four countries, where figure 2*a*,*b* demonstrates the dominant tendency of most countries, while figure 2*c*,*d* provides two examples of the reverse tendency.

**Figure 3.**
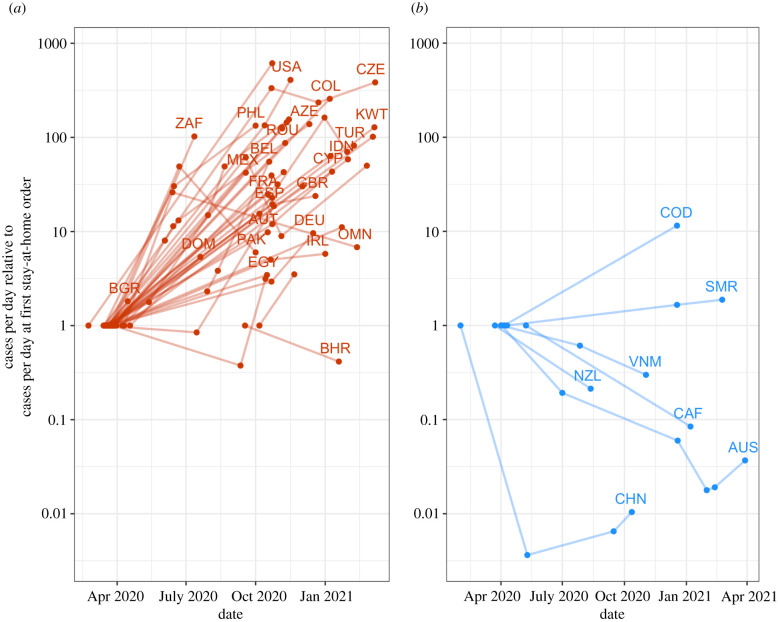
Countries implement stay-at-home orders at different levels of daily confirmed COVID-19 cases as the pandemic progresses. Note: each country is represented by a line connecting multiple dots, where each dot is the beginning of a new stay-at-home order. The position on the *y*-axis is the number of confirmed cases per day relative to the number of cases per day when that country implemented their first stay-at-home order. (*a*) Countries that have averaged more than 100 new cases reported per day since April 2020; (*b*) countries that have averaged 100 cases or fewer per day. Countries are only included if they implemented more than one stay-at-home order and had local community transmission (greater than 10 new cases per day) when they first implemented a stay-at-home order.

In figure 3, we generalize this analysis across all countries, showing how many confirmed cases there were at the point of implementation of a new stay-at-home order. This is normalized relative to the number of daily cases when each country implemented their very first stay-at-home order. In other words, the first dot for each country is always at a ‘relative cases’ value of 1, and subsequent dots represent the enactment of subsequent stay-at-home orders. Thus, a country with a trajectory rising above 1 has implemented stay-at-home orders at progressively higher daily case levels (or it has become ‘less sensitive’ to case numbers over time), and one with a trajectory dropping below 1 has become more sensitive.

Figure 3*a* includes countries with moderate or high daily transmission (averaging more than 100 confirmed cases per day from April 2020 to March 2021). In this panel, we see the general pattern where subsequent stay-at-home orders were imposed at higher case numbers than the first stay-at-home order. Notably, the countries that have exhibited the reverse tendency are almost exclusively nations that have very little transmission since April 2020—identified in figure 3*b* as countries that have averaged 100 confirmed cases or fewer per day since April 2020.

The countries exhibiting this apparent high sensitivity (implementing stay-at-home orders at progressively lower case levels) include countries that seem to have had the most success implementing the WHO's recommended ‘test, trace, isolate’ approach. For example, in the first months of 2021, Australia had several stay-at-home orders that lasted a short period of time (less than a week), for the purpose of contact tracing and isolating a new cluster [45]. These stay-at-home orders were imposed after a very small number of cases, usually less than 10. Once all contacts of the cluster were quarantined, the stay-at-home orders were lifted.

In general, these countries and regions that exhibit hyper-sensitivity have very high levels of testing and surveillance for COVID-19 outbreaks. Australia, Vietnam and New Zealand detect one positive COVID-19 case for every 500–1000 tests they perform, some of the lowest positivity rates in the world. This compares to about 1 in every 5–10 tests for Colombia, Mozambique and Indonesia, and 1 in every 10–50 for the USA, Nigeria, India and the UK. Of course, it takes many more tests and resources to achieve a low positivity rate in a country with ongoing community transmission, as the numerator (the number of confirmed, positive cases) is higher.

## 5. Policymaking moves to more granular subnational levels

The global divergence in national-level policies has been accompanied by a parallel trend of within-country variation. In some cases—such as Switzerland—the decisions behind this subnational divergence are taking place in many different independent government units, as per the federal nature of the Swiss constitution, which vests significant authority in the subnational cantons [46]. In other cases—such as Vietnam—subnational divergence has been attributed to geographically targeted policymaking from a central government [47]. In April and May 2020, almost two-thirds of all countries had a stay-at-home order in place, and around 80% of those applied the policy across the entire country. By July, fewer countries had stay-at-home orders (around one-third of all countries) and these policies were more likely to have been localized: only 40% were applied country-wide, and in stay-at-home orders in the remaining 60% of countries were targeted to specific geographical locations.

The extent of geographical targeting has, at times, been quite striking. We explore this using data from several countries for which the OxCGRT publishes subnational data: the USA, Brazil and Canada. In April 2020, 41 US states had a stay-at-home order in place—an almost completely nationwide response. By July, this had fallen to four US states, and thereafter, the country has never recorded more than one-quarter of states implementing stay-at-home orders simultaneously. Brazil's stay-at-home orders initially peaked in May 2020, when half its states (13 out of 27) had stay-at-home measures in place. For the rest of 2020, Brazil only had 5–8 states with simultaneous stay-at-home orders, but in March 2021, this rose to over 21 states. There are similar patterns in these countries for other policies like business closures.

On the surface, high levels of subnational variation might be expected. If subnational governments used consistent risk frameworks and decision rules—imposing stringent policies only in the areas most at risk—then perhaps only a few jurisdictions at any given time would have an outbreak that warrants a stay-at-home order or business closures. Results from pandemic models suggest that an agreed approach between subnational jurisdictions with sufficient policy coordination can be as effective as a nationally coordinated policy [48].

However, closer examination of the data supports widespread media reports from the aforementioned countries, suggesting that subnational policymaking has not always been driven by consistent decision frameworks [49–52]. While there appears to be a loose relationship between disease transmission and policy stringency, it is—concerningly—quite uncommon for the strictest policy in a country to be in the place with the worst outbreak. At the time of writing, there are over 450 days of policy data reported in the OxCGRT dataset (from January 2020 to March 2021). Yet, there have only been 6 recorded days where the US state with the highest rates of COVID-19 transmission also had the most stringent policies in the country (as measured by the OxCGRT stringency index), and only 13 days where the Brazilian state with the most severe outbreak had the most stringent policies. Subnational decision-making is clearly influenced by factors other than local epidemiological conditions in each state, and other researchers have begun considering factors such as political and ideological alignment of governments [53], electoral timings [54], the level of misinformation in the media [55], perceived seriousness of the pandemic and the level of institutional trust [56], and local state capacities [57].

## 6. Conclusion

COVID-19 is an era-defining, global event, for which governments have responded by enacting extraordinary policies. We have identified three broad trends in their use of NPIs during the first year of this pandemic. First, following an initial, rapid convergence in bringing in closure and containment policies for the first time, we observe gradual global divergency in stringency, with regional similarities in the maintenance of stay-at-home orders. Second, focusing on stay-at-home orders, perhaps the toughest of all NPIs for individuals to observe over time, we identify a distinction in the apparent sensitivity of countries to reintroduce these measures, with one group of countries (that had previously beaten community transmission) readily reintroducing stay-at-home orders at low case levels, while most other countries (with higher historical case rates) tended to hold back. Third, we note the growth of within-country policy divergence over time, and the infrequent association between subnational policy strength and the relative size of local outbreaks.

Our analyses allow us to describe patterns in policies enacted, but not to causally explain the rationale of decision-making. To be sure, one can imagine that during the first months of the pandemic, without clear scientific advice, a precautionary approach would have led to the high levels of stringency that we observe almost universally. The regional trends in stay-at-home orders, during a period of broader, global policy divergence, may have their origins in between-country policy learning, seasonal climatic factors, cultural views around public equity (or societal acceptance in the trading off of certain human rights against others) [58] or indeed in differing regional levels of resources for providing economic support. Similarly, although it is tempting to read path dependency into the readiness of countries to reimpose stay-at-home policies, as fits the observed pattern, we are unable to pin down a causal explanation.

This exposes a rich research agenda to elucidate the decision-making processes and logics behind the scenes, and to further explore the role of path dependency in COVID-19 pandemic policymaking. There is a litany of questions within such a research agenda. For example, to the extent that pandemic management (and crisis management in general) exhibits path dependency based on early decisions, can we identify where the critical juncture, or junctures, occurred? Moreover, how might countries (or subnational jurisdictions) change paths afterwards?

Looking ahead from the time of writing—a little beyond the 1-year mark of the pandemic—another variable has entered the scene. The rates and efficacy of massive vaccination campaigns will no doubt play an important factor in the use of NPIs going forward. Seeking to make sense of tendencies in NPI adoption while vaccination programmes spread gradually, unequally and inconsistently around the world could greatly assist policymakers in making better decisions to overcome this pandemic, and in managing of other global crises in the future.

## Data accessibility

The data used are from the publicly available Oxford COVID-19 Government Response Tracker which can be accessed at https://github.com/OxCGRT/covid-policy-tracker. An R script to produce the charts in this paper can be found here: https://github.com/OxCGRT/covid-policy-publications/blob/main/stay-at-home%20orders/PHILLIPS%20covid%20stay-at-home.R.

The data are provided in electronic supplementary material [59].
